# A narrative review on adverse drug reactions of COVID-19 treatments on the kidney

**DOI:** 10.1515/med-2023-0867

**Published:** 2024-03-15

**Authors:** Fatemeh Jahanshahi, Seyed Behnam Jazayeri, Mohammad Mirahmadi Eraghi, Leonardo Oliveira Reis, Mahtab Hamidikia, Shayan Amiri, Seyed Mohammad Kazem Aghamir

**Affiliations:** Research Committee Member, Faculty of Medicine, Iran University of Medical Sciences, Tehran, Iran; Urology Research Center, Tehran University of Medical Sciences, Sina Hospital, Tehran, Iran; Students’ Scientific Research Center (SSRC), Tehran University of Medical Sciences, Tehran, Iran; School of Medicine, Qeshm International Branch, Islamic Azad University, Qeshm, Iran; UroScience and Department of Surgery (Urology), School of Medical Sciences, University of Campinas, Unicamp, and Pontifical Catholic University of Campinas, PUC-Campinas, Campinas, São Paulo, Brazil; Rasool Akram Medical Complex, Iran University of Medical Sciences, Tehran, Iran

**Keywords:** AKI, drug-induced reaction, COVID-19, DARI, SADR

## Abstract

Studies showed that the respiratory is not the only system affected by coronavirus 2, while cardiovascular, digestive, and nervous systems, as well as essential organs such as the kidneys, can be affected by this virus. In this review, we have studied the epidemiology, clinical, and laboratory findings on COVID-19 infection renal involvement, mortality, physiopathology, remaining renal sequels after recovery, underlying renal disease, and renal injury due to its treatment. Also, protective measures for kidney injury are explained in three levels. Evidence of viral particles and genome in the urine and renal tubular cells and signs of damage such as microangiopathy, hypercoagulopathy, and fibrosis are found in COVID-19 patients. The result of this study showed, in hospitalized COVID-19 patients, that the rate of acute kidney injury (AKI) was up to 46%, with a mortality ranging from 11 to 96%. A considerable proportion of patients with AKI would remain on renal replacement therapy. Proteinuria and hematuria are observed in 87 and 75% patients, and increased Cr and glomerular filtration rate (GFR) <60 ml/min per 1.73 m^2^ are observed in 29.6 and 35.3% of the patients, respectively. Remedsivir is considered to have adverse effects on GFR. COVID-19 patients need special attention to prevent AKI. Those with underlying chronic kidney disease or AKI need proper and explicit evaluation and treatment to improve their prognosis and decrease mortality, which should not be limited to the hospitalization period.

## 1 Introduction

Since December 2019, the first case of severe acute respiratory syndrome was reported in Wuhan, China, and the coronavirus then spread increasingly worldwide and became a global dilemma [1,2,3]. SARS-CoV-2 is a leading cause of acute respiratory disorder, including interstitial and alveolar pneumonia. A significant association between SARS-CoV-2 and multiple organ failures, including the cardiovascular system, gastrointestinal tract, nervous system, and kidneys, has been established [4,5,6,7].

Our knowledge of acute kidney injury (AKI) pathophysiology in COVID-19 is expeditiously evolving. The review of the literature on COVID-19 has shown that there are several etiologies, including proximal tubular damage, prerenal azotemia, thrombotic microangiopathy, glomerulopathy, and complications secondary to the management of the disease [8,9,10]. AKI represents a cumulative health burden worldwide regarding mortality, morbidity, and economic impacts [8]. According to a meta-analysis of an international cohort conducted by Susantitaphong et al., the incidence rate of adults’ inpatient AKIs accounts for 22% with a 24% rate of pooled AKI-associated mortality [11]. Furthermore, it is increasingly identified as a predisposing factor for end-stage renal disease (ESRD) and chronic kidney disease (CKD) [12,13,14,15].

A growing body of the literature has elucidated a trend for AKI susceptibility, accompanied by a higher mortality rate in patients suffering from COVID-19. AKI may show the patient’s disease intensity and remains a survival prognosis [16]. The mortality rate of AKI associated with COVID-19 ranges from 35 to 80%; however, this rate accounts for 75 to 90% of those who require renal replacement therapy (RRT) [17]. The occurrence of AKI in severe cases represents a wide range of ratios accounting for 2.9–50% [18].

The occurrence of proteinuria or hematuria and the rise of serum creatinine represent evidence of glomerular injury in patients suffering from COVID-19 [19]. In the current literature review, the presence of proteinuria and hematuria among COVID-19 patients remains 15.5 to 87% and 26.7 to 75.8%, respectively [3,18,19,20,21,22,23,24,25]. According to the investigation of 701 COVID-19 patients, Cheng et al. asserted that at the admission serum creatinine was 14%, urea was 13%, and estimated glomerular filtration rate (eGFR) < 60 mL/min/1.73 m^2^ was 13% [25]. In another trial of 59 similar patients, it was found that the level of blood urea nitrogen was elevated in 27% of all cases [25]. Patients with AKI demonstrated an elevated level of leukocyte counts, C-reactive protein, procalcitonin, lactate dehydrogenase, and alanine transaminase. Moreover, they had elevated levels of transaminases, D-dimer, cardiac troponin I, and interleukin (IL)-6 with a significantly reduced level of lymphocytes, platelet counts, and glomerular filtration rate (GFR), when compared with non-AKI patients [18,20,26]. AKI incidence is significantly correlated with different personal characteristics such as age, gender, ethnic minorities, and financial difficulties. Owing to the aged kidneys and elevated baseline levels of serum creatinine, elderly patients suffering from COVID-19 may contribute to poor outcomes [18,26,27].

In the literature review, the median age ranged from 53.5 to 73 years with 33.3–50% male patients [18,20,26,27]. In addition, the incidence of cardiovascular diseases, chronic respiratory disease, and diabetes remains higher in AKI patients when compared with non-AKI ones. Due to the pre-COVID-19 disparities and a higher load of untreated comorbidities, racial and ethnic minorities have shown unfavorable consequences of COVID-19, including the higher occurrence of renal failure. Augmentation of economic difficulties amid the COVID-19 pandemic raises the risk of the occurrence of albuminuria and rapid kidney dysfunction. This concern leads to a higher prevalence of kidney disease as well as exceeding progression in the aforementioned population [28].

Concerning the pivotal role of kidneys in the human body, several investigations have been conducted to familiarize with the remained renal sequels after complete recovery from the infection. We hereby seek to describe the available evidence regarding the aforementioned organ in the context of COVID-19 patients [29,30].

Drug-induced acute renal injury is regarded as a kidney injury triggered by drugs or their metabolites up to 7 days after consumption. In clinical practice, it is increasingly identified as a relatively frequent adverse drug reaction (ADR). In the United States, drugs are responsible for 18–27% of all hospital-acquired AKI [31,32]. In a multicenter cross-sectional survey of Chinese hospitalized patients, 71.6% of AKIs represented the background of potential nephrotoxic drug use [33,34,35].

Although there is not yet available medication with Food and Drug Administration (FDA) approval for the treatment of COVID-19, investigators proposed remdesivir (RDV), antimicrobials, nitazoxanide, lopinavir/ritonavir, azithromycin, and ivermectin to be used during the current pandemic. Besides, host cell modulators such as chloroquine, hydroxychloroquine (HCQ), and agents affecting the host immune system have additionally been recommended. As more evidence is being provided, postulated COVID-19 therapies are garnered and lose their support after a while. The aforementioned dynamic has affected the therapeutic recommendations influencing the availability of RDV, HCQ, and convalescent plasma (CP) employing emergency use authorization (EUA) via FDA [36].

Inpatient care of COVID-19 patients reflected that the incidence rate of severe adverse drug reactions (SADR), i.e., 4.75-fold, is higher than non-COVID-19 patients. This would raise concerns when employing medications for COVID-19 patients, particularly in those agents which are well known as predisposing factors of myotoxic, hepatotoxic, and thromboembolic events [37,38].

Regarding the pathological view, edema, inflammation, and a reduced density were reported in renal tissues of the COVID-19 patients [26]. As observed through the electronic microscope and immunohistochemistry evaluations, SARS-CoV-2 nucleocapsid protein antigens are being aggregated in the renal tubules. During the second and third weeks after SARS-CoV infection, virus shedding was also detected in the urine, and collapsing glomerulopathy was observed as well [39]. Direct viral damage with or without disturbed renal hemodynamics, i.e. ischemic reperfusion injury, could yield AKI in COVID-19 [40].

Postmortem investigations demonstrated lymphocyte infiltration in renal tissues [41]. ACE2 receptors provide the main binding site for SARS-CoV-2. Brush border apical membrane of the proximal tubules, as well as podocytes, expresses ACE2 in the renal tissues, which may elucidate the cause of virus entries to the glomerular capillaries/arterioles and infection of the glomerular endothelial cells [42,43,44]. In this regard, the following underlying mechanisms for the internalization of the aforementioned virus have been proposed [39]:

1. Via binding to the host cell ACE2 receptors through SARS-CoV-2 viral spike protein: Furthermore, it requires a cellular serine protease called TMPRSS2 to cleavage the spike protein and facilitate its cell entry.
2. Via the cleavage activation of SARS-CoV-2 spike protein through cathepsin in endosomes.

ACE2 downregulation induced by the virus could decrease its anti-inflammatory role and trigger the damaging bradykinin-BKB1R axis and angiotensin II–AT1R axis leading to organ damage. AT1R activation due to viral infection provides the ACE2 soluble form and it is subsequently released into the body fluids by ADAM17 [39].

The virus attacks target cells via CD147, which is highly expressed on the renal proximal tubules and infiltrating inflammatory cells, and plays a pivotal role in several kidney damages by inducing inflammatory responses and dysregulation of the cell cycle [44,45].

The virus entry drastically downregulates ACE2 expression and, therefore, decreases anti-inflammatory functions and increases the impact of angiotensin II in affected patients. Accordingly, the binding of angiotensin II to AT1 reduces the generation of angiotensin, which subsequently yields AKI triggering, coagulation, and pulmonary inflammation [46,47].

Hyperinflammatory responses induced by SARS-CoV-2 play a crucial role in terms of AKI development, the severity of infection, and mortality [39]. Macrophages present SARS-CoV-2 antigen to CD4^+^ and Th1 cells, leading to the release of IL-12 and further activation of Th1 cells. The triggered Th1 cells promote B-cells to release more antigen-specific antibodies and CD8^+^ T cells against target cells accommodating viral antigens [48]. CD8^+^ cells impose an antiviral effect by triggering their cytotoxic effect. T cells induce pro-inflammatory cytokine production via triggering the nuclear factor-κB signaling pathway. The cytokine storm incidence secondary to the secretion of chemokine/cytokines including IL-21, IL-8, IL-6, IL-1β, tumor necrosis factor-α, and CCL-(5,3,2) accounts for multiple organ damage [44].

The cytokine storm probably contributed to the AKI in COVID-19 patients by communicating with renal resident cells as well as endothelial and tubular dysfunction. IL-6 induces kidney vascular permeability and promotes renal endothelial cells to release pro-inflammatory chemokines/cytokines, which can induce the production of thrombosis, disseminated intravascular coagulation, capillary leak syndrome, and pose microcirculatory dysfunction [49].

As cytokines can trigger macrophages, anemia, hemophagocytosis, disturbances of vascular hemostasis, and subsequent renal failure are also expected [50].

The viral infection induces the infiltration of CD68^+^ macrophages, CD4^+^ T cells, and CD56^+^ natural killer cells into the tubule-interstitium, leading to tubular damage [51]. Furthermore, infiltration of the aforementioned immune cells into the infected renal tissue may trigger apoptosis, fibrosis, and micro-vascular distribution [52,53]. The proinflammatory chemokines such as CCL2 and CCL14 play a significant role in the context of the pathogenesis of AKI via communication with ACKR2. ACKR2 circumscribes inflammation, leukocyte infiltration, and fibrotic tissue remodeling following AKI, thereby avoiding the disease progression [54,55].

Hypoxia induced by COVID-19 increases the blood viscosity directly and indirectly through HRE-dependent gene transcription by inducing thrombosis [56,57]. Thrombosis laboratory indexes, e.g. prothrombin time, partial thromboplastin time, D-dimer, and international normalized ratio, were found to be elevated. Moreover, fibrinogen levels were found to be reduced, and schistocytes and thrombocytopenia were detected in peripheral blood smears [58]. Also, the antiphospholipid antibodies that are regarded as the cause of thrombotic events are elevated in COVID-19 patients suffering thrombocytopenia [58]. Hypercoagulation may develop cortical necrosis and impose irreversible renal damage in severe COVID-19. In this regard, microangiopathy and microthrombi conditions may spread micro-infarctions to different organs including the heart, kidney, and liver [59,60].

Rhabdomyolysis may be a subsequent complication of COVID-19. In this respect, glomerular fibrin thrombi with accompanying ischemic collapse, acute proximal tubular damage, and peritubular erythrocyte aggregation were obtained from the autopsy of COVID-19 patients [61,62,63].

Regarding increasingly off-label consumption of related medicines, the COVID-19 patients in 2020 reflected a 5.8-fold higher incidence rate of SADRs when compared to the same period of 2019. Hence, the current review provides more insights into the serious ADRs of the most commonly used drugs in the context of the COVID-19 patients and AKI. Larger, well-controlled investigations evaluating the efficacy and safety of therapeutic agents for COVID-19 with accompanying kidney diseases are warranted [64,65,66].

In the current study, we have reviewed the epidemiology, physiopathology, clinical and laboratory findings on renal involvement, and remaining renal sequels after COVID-19 infection recovery. Furthermore, underlying renal disease and renal injury due to the treatment of COVID-19 patients are discussed. Protective measures for kidney injury were described in the three following levels: prevention of possible causes of AKI, improvement of the prognosis and outcomes of patients suffering AKI, and reduction of the long-term sequels of AKI.

In hospitalized COVID-19 patients, the rate of AKI is reported up to 46%, mostly affecting the elderly, with a mortality ranging from 11 to 96%. These numbers are even higher in patients with underlying renal diseases. A considerable proportion of patients with AKI would remain on RRT. Proteinuria and hematuria are observed in 87 and 75% of the patients, respectively. Moreover, in patients admitted to intensive care units, the increased creatinine and GFR <60 ml/min per 1.73 m^2^ were detected in 29.6 and 35.3% of the individuals, respectively. Limited study data regarding ADRs of COVID-19 treatments on the kidney are available. Several drugs have been included in the trials on the treatment of COVID-19. As of August 2021, RDV, HCQ sulfate, chloroquine phosphate (CQ), monoclonal antibody, bamlanivimab (LY-Cov555), baricitinib (Olumiant), casirivimab, and imdevimab are the experimental medications being used during the COVID-19 pandemic as EUA drugs, among which RDV was found to have adverse effect on GFR.

## 2 ADR of COVID-19 treatments on kidneys

In a study conducted by Ramírez et al., the incidence rate of SADRs in all detected COVID-19 patients was 760.63 per 10,000 patients (95% confidence interval (CI), 707.89–816.01). This rate was 5.84-fold more than the SADR incidence rate for non-COVID-19 patients (95% CI, 137.09–186.80, 160.15 per 10,000 patients). The SADR rate was detected for the same period in 2019 (95% CI, 109.53–154.36, 130.19 per 10,000 patients). The most notable associated medications were reported as follows: tocilizumab, dexketoprofen, azithromycin, lopinavir–ritonavir, dexamethasone, and chloroquine/HCQ [38]. From the beginning of the COVID-19 outbreak in Wuhan, till now, when global vaccination efforts are paving their path, several therapeutic regimens have been tried to find the effective treatment. However, given the steep rise in the number of patients requiring critical care globally, healthcare providers were facing an unprecedented situation that was forced to manage their patients with drugs that were not adequately evaluated [67]. Therefore, many drugs were used although they were not FDA approved. On the other hand, patients suffering from COVID-19 could be more vulnerable to drug interactions due to their subsequent co-medications or comorbidities that are underlying CKD, cardiovascular disorders, obesity, and so on [68]. In addition, the metabolism of many drugs including those used in patients with COVID-19 depends on proper renal function, which can be impaired in critically ill patients [69]. This can cause serious drug toxicities, leading to drug discontinuation or organ damage. Therefore, the knowledge of ADR of COVID-19 treatments on kidneys is important. Here, we reviewed the ADRs of candidate therapies on the kidneys. These drugs have issued an EUA or approval from the FDA for the management of COVID-19 (Table 1).

**Table 1:** EUA issued drugs and their possible adverse effects on kidneys

Drug	Dosage for COVID-19	Caution for COVID-19	Adverse drug reaction on kidney^*^	FDA description	Date of EUA issue	Reference
**Antiviral**						
Remdesivir (Veklury)	Loading dose: For patients from 3.5–40 kg body weight: 5 mg/kg For patients 40 kg and higher: 200 mg	Pediatric patients (greater than 28 days old) must have an eGFR determined and full-term neonates (at least 7 days to less than or equal to 28 days old) must have serum creatinine determined before starting veklury and be monitored during treatment as clinically appropriate. Veklury is not recommended in pediatric patients (greater than 28 days old) with eGFR less than 30 ml/min or in full term with serum creatinine greater than or equal to 1 mg/dl.	Veklury has been studied in three phase 3 studies and four phase 1 studies for patients with COVID-19.	FDA approved for use in adult and pediatric patients 12 years of age and older and weighing at least 40 kg for the treatment of COVID-19 requiring hospitalization. Remdesivir also remains authorized for emergency use for the treatment of suspected or laboratory-confirmed COVID-19 in hospitalized pediatric patients weighing 3.5 kg (about 7.7 pounds) to less than 40 kg or hospitalized pediatric patients less than 12 years of age weighing at least 3.5 kg	October 22, 2020	[118]
			No renal adverse reaction in all these studies was more than placebo group or standard of care group.			
	Maintenance dose: Patients from 3.5 to 40 kg body weight: 2.5 mg/kg For patients 40 kg and higher: 100 mg		Most common renal adverse events based onVigiaccess™ database(42): Aki (8%) Renal impairment: (2.1%) Renal failure: (1.5%) Renal tubular necrosis: (0.3%) Anuria: (0.2%)			
HCQ and CQ	—	—	Most common renal adverse drug reactions of HCQ based on Vigi access™ database: Acute kidney injury (0.4%) Renal impairment (0.2%) Renal failure (0.2%) Proteinuria (0.1%) Nephrolithiasis (0.1%)	EUA revoked	March 28, 2020 EUA issued	[58]
			Most common renal adverse drug reaction of CQ based on Vigiaccess™ database: Acute kidney injury (0.3%) Hematuria (0.2%) Chromaturia (0.2%) Renal failure (0.1%) Renal impairment (0.1%)	No longer use for COVID-19	June 15, 2020, EUA revoked.	
**Monoclonal antibody (immunomodulation)**						
Bamlanivimab (ly-cov555)	Single intravenous (iv) infusion of 700 mg.	Bamlanivimab is not authorized for use in patients: Who are hospitalized due to COVID-19, or who require oxygen therapy due to COVID-19, or who require an increase in baseline oxygen flow rate due to COVID-19 in those on chronic oxygen therapy due to underlying non-COVID-19 related comorbidity No dosage adjustment is recommended in patients with renal impairment.	No renal adverse drug reaction is reported based on studies of patients with COVID-19.	EUA authorizedfor the treatment of mild to moderate coronavirus disease COVID-19 in adults and pediatric patients (12 years of age and older weighing at least 40 kg) with positive results of direct sars-cov-2 viral testing, and who are at high risk for progressing to severe COVID-19 and/or hospitalization.	November 9, 2020	[52]
			The only reported renal adverse drug reaction of bamlanivimab based on Vigiaccess™ database: Renal impairment (1.2%)			
Baricitinib (olumiant) + remdesivir	Baricitinib Adults and pediatric patients 9 years of age and older with eGFR > 60 mL/min/1.73 m^2^ 4 mg once daily Pediatric patients 2 years to less than 9 years of age: 2 mg once daily Remdesivir 200 mg on day 1 and 100 mg once daily (via intravenous infusion) on subsequent days	Baricitinib Dosage adjustments are recommended for laboratory abnormalities, including renal impairment The dose should be reduced to 2 mg once daily in adults and pediatric patients (>9 years) with eGFR 30 to <60 mL/min/1.73 m^2^ and 1 mg once daily for 2–9 year patients The recommended dose for adult and pediatric (>9 years) patients with eGFR 15 to <30 mL/min/1.73 m^2^ is 1 mg once daily Baricitinib is not recommended for patients who are on dialysis, have ESRD, haveacute kidney injury or patients 2–9 years with eGFR 15 to < 30 mL/min/1.73 m^2^	The following renal adverse drug reactions are based on studies of patients with COVID-19 .(48) Serious AKI (1%) Non-serous AKI (3%) UTI (1%) Decrease in GFR (9.7%) Decrease in creatinine renal clearance (0.6%)	EUA authorized for the treatment of suspected or laboratory confirmed COVID-19 in hospitalized adults and pediatric patients 2 years of age or older requiring supplemental oxygen, invasive mechanical ventilation, or ECMO	November 19, 2020	[52]
Casirivimab + imdevimab	The authorized dosage is 1,200 mg of casirivimab and 1,200 mg of imdevimab administered together as a single intravenous (IV) infusion	Casirivimab and imdevimab, administered together, is not authorized for use in patients: Who are hospitalized due to COVID-19, or who require oxygen therapy due to COVID-19, or who require an increase in baseline oxygen flow rate due to COVID-19 in those on chronic oxygen therapy due to underlying non-COVID-19-related comorbidity No dosage adjustment is recommended in patients with renal impairment	No renal adverse drug reaction is reported based on studies of patients with COVID-19.	EUA authorized for the treatment of mild to moderate COVID-19 in adults and pediatric patients (12 years of age and older weighing at least 40 kg) with positive results of direct sars-cov-2 viral testing, and who are at high risk for progressing to severe COVID-19 and/or hospitalization.	November 21, 2020	[52]
Fresenius propoven 2% (propofol 20 mg per ml)	Administration rates of 0.3–4.0 mg propofol/kg bodyweight/h have been demonstrated to provide adequate sedation	Caution should be taken when treating patients with mitochondrial disease, epilepsy, and disorders of fat metabolism.	No renal adverse drug reaction is reported based on studies of patients with COVID-19.	EUA authorized to maintain sedation via continuous infusion in patients older than age 16 with suspected or confirmed COVID-19 who require mechanical ventilation in an ICU setting.	June 15, 2020	Fact sheet for health care providers emergency use authorization (EUA) of fresenius propoven 2% emulsion
		Fresenius propoven 2% should not be used in patients who are hypersensitive to peanut or soy	Most common renal adverse events based on vigiaccess™ database: Acute kidney injury (0.9%) Chromaturia (0.4%) Renal failure (0.3%) Renal impairment (0.2%) Oliguria (0.1%)			
**Plasma-rich antibody**						
COVID-19 convalescent plasma	Clinical dosing may first consider starting with one convalescent plasma unit (about 200 ml), with administration of additional convalescent plasma units based on the prescribing physician’s medical judgment and the patient’s clinical response.	COVID-19 convalescent plasma may be contraindicated in patients with a history of severe allergic reactions or anaphylaxis to plasma transfusion.	No renal adverse drug reaction is reported based on studies of patients with COVID-19.	EUA authorized for the treatment of hospitalized patients with COVID-19	August 23, 2020	[67]

*The displayed percentages are obtained from two major sources; if renal adverse drug reactions and their percentages were reported in the fact sheets for health care providers or published source of each drug, we wrote the same percentages. If not, we calculated relative frequencies by dividing the absolute number of adverse reaction reports by the total number of adverse reaction reports for each drug. The latter information is obtained from WHO pharmacovigilance database (www.vigiaccess.org) for system organ classes considered relevant to patients with COVID-19 (access date January 11, 2021).

## 3 Lopinavir/ritonavir

Well-known renal adverse effects of lopinavir/ritonavir comprise a lower GFR as well as glycosuria and proteinuria in patients with positive human immunodeficiency virus (HIV) [70]. According to the retrospective analysis, 37.8% of patients with COVID-19 revealed adverse drug events, of which 63.8% of all events were attributed to consumption of lopinavir/ritonavir [71]. Although the incidence of AKI was not increased after monotherapy of lopinavir/ritonavir in COVID-19 patients in a randomized-controlled study [72], Schneider et al. announced that combining antiviral agents metabolized by CYP3A4 with HCQ is significantly associated with a higher incidence of AKI [70].

## 4 RDV

RDV is increasingly gaining attention as a therapeutic choice for COVID-19 as of receiving EUA authorization in May 2020, howbeit the comprehensive knowledge regarding the benefits and complications remains to be elucidated [73].

RDV is an analog of adenosine nucleotide that shares broad antiviral activities with many viruses including two former members of the coronavirus family, MERS and SARS-CoV [74]. The mechanism of action of RDV is inhibition of viral RNA-dependent RNA polymerase and subsequent inhibition of viral replication [75]. RDV is the only drug that has issued FDA approval for treating patients suffering from COVID-19 who require hospitalization, both adult and pediatric patients (≥12 years, ≥40 kg). The clinical benefit of RDV in COVID-19 is its potential to shorten the median time to recovery and reduce the overall mortality rate [76,77]. Owing to the weak inhibitory role of RDV triphosphate for mammalian RNA/DNA polymerases, it is potentially regarded as a carrier of mitochondrial toxicity. Available data in COVID-19 based on a single randomized controlled trial (RCT) confirmed no evidence of the elevated risk of renal adverse events in participants randomized to receive RDV. Meanwhile, no significant renal adverse event was reported after employing RDV in an RCT of Ebola [78,79,80]. RDV was well tolerated in those who presented with severely reduced kidney function, either in AKI or CKD patients, including those on hemodialysis [78].

The National Institute of Allergy and Infectious Diseases (NIAID) in an Adaptive COVID-19 Treatment Trial (ACTT-1) compared RD with placebo in a double-blind RCT with 1062 COVID-19 patients. After 10 days, patients who received RDV had a sooner recovery than those who received placebo (median 10 vs 15 days; rate ratio for recovery, 1.29 [95% CI, 1.12–1.49]; *p* < 0.001) [81]. Furthermore, all-cause mortality after 29 days of treatment was lower in the RDV group (11.4 vs 15.2%, hazard ratio, 0.73; 95% CI, 0.52–1.03). Renal adverse reactions of RDV in this study were as follows: 18% of the patients in the RDV group had a decline in estimated GFR, 18% showed a decrease in creatinine clearance, and 15% showed a rise in creatinine, compared with 20, 24, and 15% in the placebo group, respectively. These ADRs were serious only in five patients (0.9%) of the RDV group and lead to drug discontinuation versus zero in the placebo group. Note that in the NIAID ACTT-1 study and all other studies of RDV that supported data for EUA, patients with eGFR < 30 ml/min per 1.73 m^2^, patients with severe AKI and patients with ESRD were excluded [82]. To bridge the knowledge gap regarding the safety of RD in patients with COVID-19 and renal impairment, Thakare et al. in a case series of 46 COVID-19 patients who had either AKI (*n* = 30) or ESRD (*n* = 16) evaluated the safety of RDV [83]. RDV dosage was similar to that suggested for use in COVID-19 patients (200 mg on day 1, then 100 mg/day) for a total dose of 600 mg (in 44 patients) or 1200 mg (in 2 patients). The authors reported no renal function abnormality attributable to the drug. Moreover, another observational study conducted on 48 dialysis-dependent patients with SARS CoV-2 who were given 100 mg of RDV 4 h before hemodialysis showed that two to six doses of RDV over 5–11 days is safe and well tolerated in patients requiring hemodialysis [83]. To conclude, adult patients with COVID-19 with normal renal function who receive RDV should be monitored for rare, but serious adverse renal reactions. In cases of AKI or a significant decrease in the GFR, the drug should be discontinued. There is a need for more studies evaluating the safety and tolerability of RDV in the pediatric population and patients with impaired renal function; however, a few case series on RDV use in the latter group suggest that RDV is relatively safe in this population.

On March 28, 2020, FDA issued EUA for the use of CQ and HCQ for COVID-19 treatment in adults and adolescents who were hospitalized with COVID-19 [84]. HCQ rarely develops renal adverse effects, with some reports of histomorphologic change comparable to Fabry nephropathy, i.e. phospholipidosis [85]. As of that date, scientific evidence showed promising evidence in favor of the efficacy of these drugs in acting against SARS-CoV-2 *in vitro* in terms of reducing the period of viral shedding and disease duration [86]. However, emerging evidence from the RCT showed no significant difference in terms of reducing viral shedding, mortality, or other secondary outcomes for CQ/HCQ in comparison with the standard of care alone [87]. In addition, serious cardiac adverse effects such as QT prolongation, cardiomyopathy, and cardiac arrhythmia raised issues regarding the safety profile of these drugs [88]. Eventually, on June 15, 2020, the FDA revoked the EUA for the use of CQ/HCQ in the treatment of COVID-19 [89]. Regarding renal adverse events of CQ/HCQ, these drugs seem to be relatively safe [90]. Although, a few case reports have suggested Fabry’s disease mimicking injury to podocytes with the use of CQ and/or HCQ1, which needs further evaluation [90,91].

Bamlanivimab (LY-CoV555) is a recombinant monoclonal antibody-blocking SARS-CoV-2 attachment to the human ACE2 receptor by binding to the virus spike protein [92]. On November 9, 2020, the FDA authorized EUA for the treatment of patients with mild-to-moderate COVID-19 disease and those who are at high risk of progression to a severe form of COVID-19 and/or hospitalization (including CKD patients) [93,94]. The supporting data for the dosage that have authorized EUA from the FDA (single intravenous (IV) infusion of 700 mg) is sourced from an interim analysis, demonstrating that bamlanivimab can reduce the incidence of hospitalization or emergency room visits for patients (1.6% in the LY-CoV555 group and 6.3% in the placebo group) [95]. According to FDA’s fact sheet for the EUA, no kidney-related ADR has been observed regarding the use of bamlanimivab in COVID-19 trials. Furthermore, given that the drug is not excreted intact by the kidneys, bamlanivimab remains safe for use in eligible patients, including those suffering from renal impairment or CKD.

Similar to bamlanivimab, casirivimab and imdevimab are also recombinant neutralizing human antibodies. These antibodies block the receptor-binding domain of SARS-CoV-2 to the human ACE2 receptor [82]. On November 21, 2020, the combination therapy of these two drugs was granted EUA for use in patients with similar criteria to bamlanivimab EUA [93]. The supporting data for the dosage that has authorized EUA from the FDA (1,200 mg of casirivimab and 1,200 mg of imdevimab administered together as a single IV infusion) are sourced from the R10933-10987-COV-2067. This trial showed that viral load in the treatment arm changed faster in comparison with the placebo arm; the time-weighted average change from day 1 to day 7 was −0.36 log10 copies/mL (*p* < 0.0001) compared to placebo. Furthermore, patients treated with casirivimab and imdevimab had fewer medical visits for COVID-19 (2.8% for combined treatment arms vs 6.5% placebo) [96]. Based on the FDA’s fact sheet for the EUA, no kidney-related ADR has been observed for the use of casirivimab and imdevimabin COVID-19 trials [97]. Like bamlanivimab, these two drugs are not excreted intact in the urine; they, therefore, are expected to be safe in eligible patients suffering renal impairment.

Baricitinib is a selective inhibitor of Janus kinase 1 and 2 (JAK1 and JAK2), which shares immunomodulatory and anti-inflammatory mechanisms to act against the SARS-CoV-2 by preventing the phosphorylation of signal transducers and activators of transcription, which are needed for the production of granulocyte-macrophage colony-stimulating factor and pro-inflammatory cytokines such as IL-2, IL-6, IL-10, and interferon-γ [93]. On November 19, 2020, FDA issued EUA for baricitinib in combination with RDV for the management of hospitalized adults and pediatric patients (>2 years) requiring supplemental oxygen, invasive mechanical ventilation, or extracorporeal membrane oxygenation (ECMO) [93]. The supporting data for EUA authorization are sourced from the ACTT-2 trial, which evaluated the safety and efficacy of combination therapy of baricitinib (4-mg daily) for 2 weeks plus RDV (intravenously, 200 mg loading dose, and 100 mg maintenance) for 10 days in COVID-19 patients. The study showed that combination therapy of baricitinib and RDV was superior to RDV alone in treating COVID-19 markedly among patients receiving high-flow oxygen or noninvasive mechanical ventilation. Two findings supported this conclusion in the ACTT-2 trial: The first one, receiving noninvasive ventilation or high-flow oxygen who were assigned to combination therapy, was recovered 8 days sooner than the control group. The median time to recovery was 10 days in the combination group and 18 days in the control group, and the rate ratio for recovery was 1.51 (95% CI, 1.10–2.08). The second one was recovered after 15 days of treatment, and this subgroup of patients tended to reflect a better clinical status according to an eight-category ordinal scale (odds ratio, 2.2; 95% CI, 1.4–3.6) [88]. Regarding the efficacy and safety of this combination therapy in patients with renal impairment, it is important to note that the ACTT-2 trial did not enroll patients suffering from AKI or ESRD. The FDA also does not recommend this combination therapy for patients who are on dialysis, have ESRD (eGFR <15 mL/min/1.73 m^2^), or have AKI. According to the supplementary material of the ACTT-2 study, 71 of 507 (14%) patients who received combination therapy and 75 of 509 (14.7%) patients who received RDV alone experienced nonserious renal adverse effects including a decrease in GFR or creatinine renal clearance and an increase in blood creatinine and AKI. Spontaneous renal artery dissections including serious AKI or renal failure occurred in 1 and 2.2% of patients on the combination arm and control group, respectively. To conclude, observed clinical benefits of baricitinib plus RDV in the ACTT-2 study need further evaluation of inpatients suffering from renal impairment. Close monitoring of renal function in the patient is mandatory during administrating baricitinibin combination with RDV.

COVID-19 CP is an antibody-rich plasma obtained from COVID-19 patients who have recovered. The use of CP in the treatment of infectious diseases has a history of more than a century with the notion of “passive immunization” in the mind [98]. On August 23, 2020, FDA issued an EUA for the use of COVID-19 CP for the treatment of COVID-19 patients who required hospitalization [99,100]. The authorization was based on the safety and efficacy of CP in previous outbreaks of other coronaviruses; for example, small case series during the prior MERS-CoV and SARS-CoV outbreaks suggested using CP in the patients remains safe and efficacious in boosting viral clearance especially if administrated early at the onset of the disease [101]. In the announcement of EUA, the FDA warned about the lack of prospective RCT for the use of CP in COVID-19 patients and stated that data suggest that the use of COVID-19 CP with high antibody titer may be effective in reducing mortality in hospitalized patients with COVID-19 [100]; however, studies evaluating the efficacy of CP in COVID-19 patients have been inconclusive. An RCT in 334 patients with severe COVID-19 pneumonia showed that a total anti-SARS-CoV-2 antibody titer of at least 1:800 has no significant clinical benefit as compared with placebo [102]. The latest findings on the use of CP in COVID-19 patients are based on a retrospective study from Mayo’s clinic expanded-access program [103,104]. In this study, patients were categorized into three groups with high, medium, and low IgG antibody levels, and the primary outcome was mortality after 30 days of transfusion. The author found two promising findings: First, only for a subgroup of patients who had not received mechanical ventilation, high-titer of CP was associated with a lower risk of death within 30 days than in the low-titer group (relative risk 0.66 [95% CI, 0.48–0.91]); however, there was no difference in the risk of mortality for patients who received mechanical ventilation (relative risk, 1.02; 95% CI, 0.78–1.32). Second, transfusion of CP within 3 days of diagnosis was associated with a lower risk of mortality in comparison with patients who received CP, 4 or more days after diagnosis. In other words, the point estimate of the mortality rate was 22.2% (95% CI, 19.9–24.8) in <3 days group compared with 29.1% (95% CI, 27.6–31.6) in 4 or more days group. These findings suggest that benefits of CP in COVID-19 are most apparent in the subgroup of patients not receiving mechanical ventilation early (<3 days) in the course of the disease. However, prospective randomized studies are still most welcome in this debate. Regarding the safety profile of the CP, the Expanded Access Program has studied 20,000 patients and did not find any transfusion-related adverse events in kidneys [105]. The limitation of the current study is that novel studies are being published during the review process of our study, which might be inconsistent with previous studies and ours where by it can be searched and modified again in the final proof.

Limited documented data were found regarding the aforementioned medications in patients suffering renal impairment with accompanying COVID-19 or other indications.

## 5 Other potential nephrotic agents

Contrast-induced nephropathy (C-IN) represents a well-known etiology AKI, accounting for approximately 11% of all AKIs in hospitalized patients. It also accounts for the third most frequent etiology of iatrogenic kidney injury [106,107]. Although poorly understood, multifactorial etiologies are responsible for C-IN, including free radical-mediated direct nephrotoxicity, cellular apoptosis, and renal medullary hypoxia [108,109]. Preexisting renal involvement is considered the leading predisposing factor for CA-AKI development [110]. However, using iodinated contrast was tempered as concerns regarding the worsening of COVID-associated AKI. This theory may be mediated by multiple agents, e.g., complement activation, direct renal viral infection, microangiopathy, and thrombosis-associated ischemia [110].

A small documented series demonstrate the elevated rates of AKI patients undergoing coronary catheterization and CTA [111,112]. Sedaghat et al. [110] retrospectively evaluated the risk for CA-AKI among newly detected COVID-positive subjects without eGFR > 30. The risk of IV iodine-associated renal injury was quantified by comparing COVID-positive cases undergoing CTA and noncontrast computed tomography. A total of 258 renal-competent subjects with eGFR > 30 with baseline and 48- to 72-h postexamination were recruited. Twenty-five of 191 patients undergoing CTA and 9 of 67 patients undergoing noncontrast CT increased Cr profile meeting the criteria for developing CA-AKIs. No positive correlation was achieved between the criteria meeting CA-AKI and iodinated contrast administration.

A retrospective study investigating the epidemiology and tolerability of nephrotoxicity-related profile of conventional amphotericin B administration among 117 patients was conducted by Abdel‑Hafez et al. [113]. They excluded all patients younger than 12 years, those who received fewer than two doses, the drug intermittently, through non-IV routes. The data collection was not limited to gender, age, amphotericin B treatment details, comorbidities, medications, COVID-19 status, and hypothesized protective measures. The incidence rates for hypokalemia and conventional amphotericin B-associated nephrotoxicity were 33 and 46%, respectively.

The median age of patients was 52 years and the range was 13 to 89 years, and a roughly equal sex ratio was also identified. No positive correlation between the nephrotoxicity development and the variables was detected. However, a significant 3.4 increased risk was observed in females rather than males in the context of developing hypokalemia.

Romaní et al. [114] performed a cross-sectional analytical study to determine the underlying association between AKI development and antibiotics administration among patients suffering from SARS-CoV-2. Vancomycin was found to be meaningfully associated with AKI development. The drugs most frequently applied for COVID-19 management were: vancomycin, aminoglycosides, ivermectin, tocilizumab, azithromycin, and corticosteroids, among which vancomycin administration was found to be related to AKI development among hospitalized subjects. Vancomycin-related nephrotoxicity was conventionally attributed to impurities included during the process of antibiotic preparation. Improved fermentation methods have significantly reduced the potential toxicity. However, vancomycin-induced renal toxicity is manifested in 10–20% of patients [115,116].

In a retrospective observational study, Almehmadi et al. [115,117] assessed renal function using urinalysis in patients with a free kidney disease suffering from COVID-19 following vancomycin administration. They enrolled 227 patients, of whom 147 were males; 33.48% were nondiabetic; 11.89% were prediabetic; and 54.63% were diabetic. Proteinuria, glycosuria, hematuria, ketonuria, and coinfection were identified among all subjects. The mortality rate among their study group was reported to be 16.2%, of which 6.6% had vancomycin administration and 9.6% had no vancomycin administration. They found no meaningful correlation between abnormalities and nephrotoxicity in the mortality rate and urine. The abnormal finding of vancomycin group urinalysis was less common than others, except for the prediabetic participants.

## 6 Conclusion

We conclude that it is essential to use proper treatments according to the causes of kidney injury. Further clinical studies are warranted to establish the possible practical treatment for AKI patients suffering from COVID-19. In addition, COVID-19 patients at higher risk need special attention to prevent the occurrence of kidney injury. Patients with underlying CKD or new-onset kidney injury need proper and explicit evaluation and treatment to improve their prognosis and decrease mortality. Eventually, evaluations should not be limited to the hospitalization period but should be followed by regular kidney function evaluations.
